# Association of Personality with Cognitive Failure among Japanese Middle-Aged and Older Adults

**DOI:** 10.3390/ijerph19127215

**Published:** 2022-06-12

**Authors:** Hajime Iwasa, Yuko Yoshida, Yoshiko Ishioka, Yoshimi Suzukamo

**Affiliations:** 1 Department of Public Health, Fukushima Medical University School of Medicine, Fukushima 960-1295, Japan; 2 Tokyo Metropolitan Institute of Gerontology, Tokyo 173-0015, Japan; yossy@tmig.or.jp; 3 Jindal School of Liberal Arts and Humanities, O.P. Jindal Global University, Sonipat 131001, Haryana, India; ymishioka@jgu.edu.in; 4 Graduate School of Medicine, Tohoku University, Sendai 980-8575, Japan; suzukamo@med.tohoku.ac.jp

**Keywords:** cognitive failure, conscientiousness, middle-aged and older adults, neuroticism, personality

## Abstract

This study explored the associations between personality traits and cognitive failure (including minor lapses and prospective and retrospective memory failure) among middle-aged and older adults living in Japan. The participants were 373 adults, aged 40–84 (167 men and 206 women). The 15-item Japanese version of the Short Inventory of Minor Lapses was used to evaluate minor lapses, and the 16-item Japanese version of the Prospective and Retrospective Memory Questionnaire was used to assess prospective and retrospective memory failure. The participants’ variables evaluated for their association with cognitive failure were gender, age, education, paid work, social network, chronic disease, sleep quality, and the Big Five personality traits (i.e., neuroticism, extraversion, openness, agreeableness, and conscientiousness). Multivariable regression analyses demonstrated that sleep quality (β = −0.232), neuroticism (β = 0.163), and conscientiousness (β = −0.295) were related to minor lapses; age (β = 0.152), sleep quality (β = −0.168), and conscientiousness (β = −0.290) were associated with prospective memory failure; and age (β = 0.268), sleep quality (β = −0.146), and conscientiousness (β = −0.221) were associated with retrospective memory failure. These findings may facilitate the development of efficient strategies for the prevention of cognitive dysfunction and its adverse consequences for personal health.

## 1. Introduction

With the aging population increasing worldwide, the number of older adults with neurocognitive disorders, including dementia, is expected to increase in Japan. In 2012, the number of older adults with dementia was approximately 4.62 million (a prevalence of 15%); this is projected to reach 6.75 million (18.5%) in 2025 and 7.97 million (21.1%) in 2050 [1]. As individuals with dementia often present cognitive disorders (memory disorders, disorientation, and visuospatial cognitive impairment) as core symptoms, countermeasures in the health, medical, welfare, and long-term care sectors are needed. Meanwhile, cognitive function declines gradually, even with normal aging. Therefore, knowledge of the nature of normal decline in cognitive function could distinguish dementia, which involves abnormal aging, from normal aging, in terms of cognition [2] and contribute to the development of future countermeasures (i.e., screening, prevention, and welfare measures for dementia). 

Recent research has indicated that the subjective evaluation of cognitive function could be used to predict the future onset of dementia [3]. Useful information for the development of countermeasures against neurocognitive disorders could be obtained by investigating the characteristics related to the subjective evaluation of cognitive function in middle-aged and older adults and by exploring its associated factors. Presently, an increasing number of studies have examined the characteristics of the subjective evaluation of cognitive function [3,4,5,6,7,8,9,10,11,12]. Cognitive failure is a form of subjective evaluation of cognitive function. 

Cognitive failure is the occurrence of cognition-based slippage and lapses in simple operations that an individual can normally accomplish without error (e.g., leaving things behind and missing appointments) in daily living spaces, such as homes and workplaces [7,8,9]. Investigating a variety of factors associated with cognitive failure may help in identifying countermeasures against it. Several factors relating to cognitive failure have been investigated in healthy adults [13], including low cognition (i.e., attention and memory function) [14], memory complaints [15], fatigue [10,16], sleep disorders [17], daytime sleepiness [18], emotional support [10], and personality [7,10,19]. 

This study focused on personality as a factor associated with cognitive failure. Personality is defined as the “psychological qualities that contribute to an individual’s enduring and distinctive patterns of feeling, thinking, and behaving” [20]. One popularly used model is the Big Five model, which includes five major domains of personality traits: neuroticism, extraversion, openness, agreeableness, and conscientiousness [21]. High vulnerability to psychological distress is referred to as “neuroticism”; a disposition to be sociable and have positive emotional experiences is explained by the term “extraversion”; a propensity for intellectual curiosity and a preference for varied experiences is characterized as “openness”; “agreeableness” is defined as a trusting, caring, and cooperative temperament; and “conscientiousness” is characterized as a hardworking, organized, and achievement-oriented trait. As personality has been shown to determine health-related behaviors [22], attention to the relationship between personality and cognitive failure may provide useful findings for the development of countermeasures. Although some studies have indicated that personality traits are associated with cognitive failure [7,10,19], findings are limited. In particular, few studies have examined these associations among middle-aged and older adults as representative community-living groups. In addition, results obtained from diverse populations are lacking. The current study participants are highly diverse in terms of age, gender, education, employment status, social network, and health status. Identifying personality traits associated with cognitive failure could help identify those prone to cognitive failure and ascertain future countermeasures for neurocognitive disorders. 

Against this backdrop, this study investigated the association between personality traits and cognitive failure (including minor lapses and prospective and retrospective memory failure) among middle-aged and older adults in Japan.

## 2. Materials and Methods

### 2.1. Participants

The participants of this study were middle-aged and older adults, between the ages of 40–84, living in a community in Tokyo. Using a stratified, two-stage random sampling method, 1200 people were selected as prospective participants. In the first stage, 30 sites were selected from municipalities in the Tokyo metropolitan area. In the second stage, 40 people per location were randomly selected from the municipality’s residential registration files. The survey was conducted between October and November 2020 using a postal questionnaire method. First, the survey instructions and the questionnaire form were mailed to the participants. Subsequently, the participants read the instructions, answered the survey questionnaire anonymously, and submitted their responses via mail. 

### 2.2. Measures

#### 2.2.1. Cognitive Failure

This study used two scales to measure cognitive failure, including minor lapses and memory failure. Minor lapses are comprehensive indicators of cognitive failure. Prospective and retrospective memory failures are indicators specific to memory lapses. The Japanese version of the Short Inventory of Minor Lapses (SIML) was used to measure minor lapses [9,23]. This scale comprises 15 items that assess the frequency of minor lapses in daily life (e.g., “How often do you forget to say something you were going to mention?”). The items are evaluated on a five-point Likert scale from 1 (rarely) to 5 (almost always); the responses to the 15 items were combined to produce a score of minor lapses, ranging from 15 to 75 points. The higher the score, the greater the degree of minor lapses (Cronbach’s alpha coefficient in this study = 0.903). Psychometric properties of the scale have been fully examined in previous works (including factor structure, reliability, and construct validity of the scale) [9,23]. The score for minor lapses was log-transformed to reduce its skewness and was used in the regression analysis. 

The Japanese version of the Prospective and Retrospective Memory Questionnaire (PRMQ) [11,12] comprises 16 items that assess the frequency of memory failure experiences in daily life. This scale consists of two components: prospective memory failure and retrospective memory failure [24]. The two components include eight items each (e.g., “Do you decide to do something and in a few minutes time, forget to do it?” and “Do you fail to recognize a place you have visited before?”). The items are rated on a five-point Likert scale from 1 (never) to 5 (very often). The responses to the eight items in each subscale were combined to produce the scores of prospective and retrospective memory failures, ranging from 8 to 40 for each subscale. The higher the score, the higher the degree of memory failure (Cronbach’s alpha coefficients in this study were equal to 0.873 and 0.858 for prospective memory failure and retrospective memory failure, respectively). Psychometric properties of the scale have been fully examined in previous works (including reliability and construct validity of the scale) [11,12]. 

#### 2.2.2. Personality Traits

The Japanese version of the Ten-Item Personality Inventory (TIPI-J) [25,26] was used to assess the Big Five personality traits of neuroticism, extraversion, openness, agreeableness, and conscientiousness. Participants were required to evaluate each of the 10 items on a seven-point Likert scale, ranging from 1 (strongly disagree) to 7 (strongly agree). For each personality trait, the average of the two items was computed to obtain a score from 1 to 7. The higher the score, the higher the level of that personality trait. Psychometric properties of the scale have been fully examined in previous works (including reliability, correlations between the two items included in the traits, and concurrent validity of the scale) [25,26]. 

#### 2.2.3. Covariates

Participants’ age, gender, educational attainment, paid work, social network, chronic disease, and self-rated sleep quality were used as potential confounders to adjust for the independent relationship between personality and cognitive failure. Educational attainment was measured by the number of years of education the respondents had completed in their youth. Participants were questioned on their paid work status. The Japanese version of the Lubben Social Network Scale-6 (LSNS-6) [27,28] was used to determine the social network of participants. This scale is a six-item, self-report questionnaire that assesses the number of individuals with whom one is in regular contact and seeks support from. The sum of the scores for the six items was calculated to obtain a score from 0–30, with higher scores indicating a higher level of social networking. Cronbach’s alpha coefficient in this study was 0.857. Chronic disease was delineated as having one or more symptoms of stroke, heart disease, diabetes, and cancer, which were self-reported by the participants. Sleep quality was self-rated by the participants according to four values: very dissatisfied, quite dissatisfied, slightly dissatisfied, and satisfied. Higher scores indicated better sleep quality.

### 2.3. Statistical Analysis

First, study participants’ descriptive statistics were established. Second, statistical values (including skewness, kurtosis, mean, median, and standard deviation) assessing the outcome measures’ score distribution and the Pearson correlation coefficients among the outcomes were calculated. Third, multivariable linear regression analyses were performed to determine the factors associated with cognitive failure (model A for minor lapses, model B for prospective memory failure, and model C for retrospective memory failure). Participants’ age, gender, educational attainment, paid work, social network, chronic disease, self-rated sleep quality, and the five personality traits were simultaneously input into the models. To check for multicollinearity, variance inflation factors (VIFs) were computed. Post hoc power calculations for multivariable regression analyses were conducted. All probability values were two-tailed, and the significance level for all statistical tests was set at 5%. SPSS Statistics version 25 (IBM Corp., Armonk, NY, USA) was used for all statistical analyses and G*Power 3.1 [29] was used for post hoc power calculation. 

### 2.4. Ethical Considerations

This study was approved by the Ethics Committee of Fukushima Medical University (approval number: 30044, approved on 22 June 2018). All the participants were informed about the study, and it was explained that their participation was completely voluntary, they could withdraw their participation at any time without facing any penalty, and no personally identifiable information would be gathered from the survey. The participants’ act of returning completed questionnaires was inferred as their agreement with the purpose of the study and their consent for voluntary participation.

## 3. Results

A total of 409 questionnaires were received (response rate: 34.1%). Of these, 36 were excluded because of missing data on minor lapses (n = 9), prospective memory failure (n = 1), personality items (n = 5), education (n = 3), social isolation (n = 3), chronic illness (n = 14), and self-rated sleep quality (n = 1). Ultimately, the data of 373 participants (167 men and 206 women) were analyzed in this study. Table 1 shows the characteristics of the participants.

Descriptive statistics for the three outcomes (i.e., minor lapses measured by the SIML, prospective memory failure, and retrospective memory failure measured by the PRMQ) are shown in Table 2. Pearson correlation coefficients among the outcomes were as follows: the relationship between log minor lapses and prospective memory failure was 0.715 (*p* < 0.001); between log minor lapses and retrospective memory failure was 0.677 (*p* < 0.001); and between prospective memory failure and retrospective memory failure was 0.829 (*p* < 0.001). 

Multivariable linear regression analyses were performed to examine the factors associated with outcomes (Table 3). Regression model A statistically significantly predicted log minor lapses (F (12, 360) = 7.356, *p* < 0.001, an adjusted R-squared coefficient of 0.170, and a standard error of estimate of 0.244). The residuals were normally distributed, as assessed visually by a normal P-P plot. The VIF was less than two for all the independent variables, which indicated that there was no possibility of multicollinearity. Linear relationships between independent variables and minor lapses were confirmed visually by assessing a scatter plot. The results showed that sleep quality (β = −0.232, *p* < 0.001), neuroticism (β = 0.163, *p* = 0.004), and conscientiousness (β = −0.295, *p* < 0.001) were statistically significantly associated with log minor lapses. 

Regression model B statistically significantly predicted prospective memory failure (F (12, 360) = 5.512, *p* < 0.001, an adjusted R-squared coefficient of 0.127, and a standard error of estimate of 4.234). The residuals were normally distributed, as assessed visually by a normal P-P plot. The VIF was less than two for all the independent variables, which indicated that there was no possibility of multicollinearity. Linear relationships between independent variables and prospective memory failure were confirmed visually by assessing a scatter plot. The results showed that age (β = 0.152, *p* = 0.012), sleep quality (β = −0.168, *p* < 0.001), and conscientiousness (β = −0.290, *p* < 0.001) were statistically significantly associated with prospective memory failure.

Regression model C statistically significantly predicted retrospective memory failure (F (12, 360) = 4.720, *p* < 0.001, an adjusted R-squared coefficient of 0.107, and a standard error of estimate of 4.137). The residuals were normally distributed, as assessed visually by a normal P-P plot. The VIF was less than two for all the independent variables, which indicated that there was no possibility of multicollinearity. The linear relationship between independent variables and retrospective memory failure was confirmed visually by assessing a scatter plot. The results showed that age (β = 0.268, *p* < 0.001), sleep quality (β = −0.146, *p* < 0.005), and conscientiousness (β = −0.221, *p* < 0.001) were also statistically significantly associated with retrospective memory failure. Post hoc power calculations for the multivariable regression analyses were conducted for each model. In model A (sample size = 373, number of predictors = 12, alpha level = 0.05, adjusted R-squared = 0.170 (corresponding to the effect size of f-squared as 0.205)), the power (1-beta) was obtained as 0.999. In model B (sample size = 373, number of predictors = 12, alpha level = 0.05, adjusted R-squared = 0.127 (corresponding to the effect size of f-squared as 0.145)), the power (1-beta) was obtained as 0.999. In model C (sample size = 373, number of predictors = 12, alpha level = 0.05, adjusted R-squared = 0.107 (corresponding to the effect size of f-squared as 0.119)), the power (1-beta) was obtained as 0.999.

## 4. Discussion

This study investigated the factors associated with cognitive failure among middle-aged and older adults in Japan. Multivariable linear regression analyses demonstrated that sleep quality, neuroticism, and conscientiousness were associated with minor lapses; age, sleep quality, and conscientiousness were associated with both prospective and retrospective memory failures. The results of the power calculations show that the statistical power for each model was sufficient to examine the relationships between the 12 predictors and cognitive failure.

Among personality traits, neuroticism had an independent effect on minor lapses when the other variables were statistically controlled. Participants with higher levels of neuroticism exhibited more frequent minor lapses, which is similar to previously reported results [7]. According to the Big Five Model of Personality [21], neuroticism causes a proneness to experience psychological distress, and those with high neuroticism are highly likely to experience negative emotions such as depression, anxiety, and anger. Research has also reported an association between negative emotions and cognitive failure [30]. Two mechanisms have been discussed in the literature to interpret this relationship. First, persons with high neuroticism are prone to thinking negatively about the events they experience and, thus, may report greater failures and judge such events highly negatively. Second, people with high neuroticism are more likely to worry and become upset about everyday matters [21]. Therefore, they become distracted and are less able to pay attention to their daily tasks; consequently, they are susceptible to cognitive failure.

Conscientiousness had an independent effect on minor lapses and prospective and retrospective memory failures when the other variables were statistically controlled. Conscientiousness was inversely correlated with minor lapses and prospective and retrospective memory failures, which is in concordance with previous studies [10,12]. These results suggest that conscientious people tend to experience low levels of suffering from cognitive failure. Conscientiousness is a temperament, and conscientious individuals are characterized as diligent, self-controlled, organized, and achievement-oriented [21]. People who are highly conscientious may have a “cognitive-behavioral style” that is better suited to dealing with tasks in daily life [31]. For example, they may have little opportunity to experience cognitive failures because they are highly likely to organize their living environment and manage their schedules better, and they are less likely to waste attentional resources when handling routine tasks. In addition, conscientious people tend to remember important arrangements because they get their work done without any delay. Finally, they tend to easily avoid daily frustrations because they plan for the future in the long term; for example, they carefully prepare for essential tasks or future risks, such as by taking out life insurance [32]. 

The results of the regression analysis revealed that sleep quality had an independent effect on minor lapses and prospective and retrospective memory failures when the other variables were statistically controlled; that is, the higher the participants’ sleep quality, the lower their frequency of cognitive failure. Previous studies have shown that a strong relationship exists between cognitive failure and sleep disturbances [17] and cognitive failure and daytime sleepiness [18]. The findings of this study show similar results. A meta-analytic study [33] has also reported a close relationship between sleep problems (such as sleep complaints, sleep disturbances, and daytime drowsiness) and cognitive performance (including attention, working memory, episodic memory, and executive function). Such deficits in cognitive function tend to be experienced as cognitive failures (i.e., minor lapses and prospective and retrospective memory failures) in daily life among individuals who suffer from sleep problems. Thus, poor sleep quality may be associated with increased risk of cognitive failure.

The results of the regression analysis also revealed that age had an independent effect on prospective and retrospective memory failures when the other variables were statistically controlled. It is well-known that memory function declines gradually with age [2]. In addition, retrospective memory is more likely to be affected by the aging effect than prospective memory [34]. The present findings may reflect this property of memory function. Meanwhile, age was not significantly associated with minor lapses. This suggests that minor lapses may be less sensitive to the effect of aging when compared to memory failures. Therefore, future research should examine the effect of aging on minor lapses using a wider age range of participants.

This study has several limitations. First, the sample might be limited in terms of representativeness, given the low response rate (34.1% = 409/1200) and the low proportion of data collected from individuals included in the analysis (31.1% = 373/1200); therefore, the health characteristics of the participants may differ from the non-participants due to self-selection bias [35]. Second, for variables such as sleep quality and personality, an inverse relationship might exist for the assumed causal relationship. This study postulated that individuals with high scores on certain personality traits (e.g., conscientiousness) would have low levels of cognitive failure; however, the opposite may be true. Individuals with low levels of cognitive failure may have high personality scores (e.g., conscientiousness). As personality is reported to be relatively stable in adulthood [36], and there may be little scope for personality scores to change in the short term, it would be difficult to assume that cognitive failure is a cause and personality is an outcome. Because of the possibility of such a bidirectional relationship between variables, further longitudinal studies should be conducted to clarify this point. Third, the current study failed to eliminate people with neurocognitive disorders (e.g., dementia) from its participant group because information regarding the participants’ diagnostic status was unavailable. Therefore, since the participant group may include people with neurocognitive disorders, it is possible that the responses for self-rated variables may be imprecise. Fourth, the effect sizes for the individual factors and cognitive failure were relatively small to medium, even though the effect sizes were statistically significant. Thus, the findings need to be considered carefully. Fifth, this study used the TIPI to measure the Big Five personality traits. However, the NEO Personality Inventory (NEO-PI) is the most well-known questionnaire for measuring the Big Five personality traits. The Revised NEO-PI (NEO-PI-R) contains 240 items, while its short version, the NEO Five-Factor Inventory (NEO-FFI), comprises 60 items. Meanwhile, the TIPI comprises 10 items for measuring the five personality traits. As mentioned above, its psychometric properties have previously been examined; however, as the TIPI measures each factor using only two items, it may be considered to suffer from various limitations, such as providing a limited assessment of the Big Five facets. Therefore, future studies could use more comprehensive Big Five inventories (e.g., the NEO-PI-R [37]) to assess the different facets of the Big Five and their relation to cognitive failure. Finally, the present study measured sleep quality using a single item because of limited space in the questionnaire used in the study; however, it is considered desirable to measure sleep quality using a scale (e.g., Athens Insomnia Scale [38]). 

## 5. Conclusions

This study examined the factors associated with cognitive failure among middle-aged and older adults living in a community in Japan. Regression analysis indicated that age, sleep quality, neuroticism, and conscientiousness were associated with cognitive failure. Accordingly, the authors propose the following countermeasures to reduce cognitive dysfunction. First, cognitive-behavioral styles that individuals with high conscientiousness seem to possess, including excellent organizational and management capabilities, a commitment to proactively address problems, and long-term planning, can reduce cognitive failure. Second, social support from acquaintances and family members is needed to lessen the negative effects of high neuroticism (i.e., higher distress and the resulting cognitive failure). Third, it is essential for individuals to reduce fatigue by getting enough sleep and rest. As sleep disturbances are a common problem for middle-aged and older adults, appropriate coping strategies are expected to curb sleep disturbances [39,40] and consequently, cognitive failures.

## Figures and Tables

**Table 1:** Characteristics of participants (N = 373).

Gender (women), n (%)	206 (55.2)
Age, mean (SD)	61.9 (12.1)
Paid work, n (%)	247 (66.2)
Number of years of education, mean (SD)	14.3 (3.0)
Social network, mean (SD)	14.4 (6.0)
Chronic disease, n (%)	65 (17.4)
Sleep quality, mean (SD)	1.9 (0.8)
Neuroticism, mean (SD)	3.7 (1.2)
Extraversion, mean (SD)	4.4 (1.3)
Openness, mean (SD)	4.2 (1.3)
Agreeableness, mean (SD)	5.3 (0.9)
Conscientiousness, mean (SD)	4.5 (1.1)

**Table 2:** Descriptive statistics for outcome measures (N = 373).

	Minor Lapses ^a^	Prospective Memory Failure	Retrospective Memory Failure
Mean	27.06 (3.26)	16.34	14.77
Standard deviations	7.82 (0.27)	4.53	4.38
Median	26 (3.26)	16	15
Skewness	1.272 (0.476)	0.222	0.484
Kurtosis	2.134 (0.027)	−0.156	0.419

^a^ Values in parentheses are logarithmic transformations performed.

**Table 3:** Association between personality traits and cognitive failures (multivariable regression analyses) (N = 373).

	Dependent Variables											
	Model A: Minor Lapses ^a^				Model B: Prospective Memory Failure				Model C: Retrospective Memory Failure			
Independent Variables	B	SE	Beta	*p*	B	SE	Beta	*p*	B	SE	Beta	*p*
Gender	−0.002	0.028	−0.003	0.948	0.322	0.483	0.035	0.505	−0.424	0.471	−0.048	0.369
Age	0.001	0.001	0.040	0.493	0.057	0.023	0.152	0.012	0.097	0.022	0.268	<0.001
Number of years of education	0.001	0.004	0.000	0.998	−0.008	0.076	−0.005	0.919	−0.062	0.074	−0.042	0.407
Paid work	0.008	0.032	0.015	0.797	−0.813	0.560	−0.085	0.147	−0.763	0.547	−0.083	0.164
Social network	−0.001	0.002	−0.017	0.745	−0.037	0.040	−0.050	0.349	−0.024	0.039	−0.033	0.540
Chronic disease	−0.003	0.035	−0.005	0.924	−0.248	0.611	−0.021	0.685	−0.452	0.597	−0.039	0.449
Sleep quality	−0.081	0.017	−0.232	<0.001	−0.998	0.301	−0.168	<0.001	−0.836	0.294	−0.146	0.005
Neuroticism	0.037	0.013	0.163	0.004	0.307	0.219	0.081	0.162	0.272	0.214	0.074	0.205
Extraversion	−0.002	0.011	−0.011	0.835	−0.073	0.193	−0.021	0.704	−0.164	0.189	−0.049	0.387
Openness	0.020	0.011	0.094	0.084	0.053	0.200	0.015	0.790	0.025	0.195	0.007	0.897
Agreeableness	−0.009	0.015	−0.030	0.569	−0.146	0.262	−0.030	0.579	−0.092	0.256	−0.019	0.721
Conscientiousness	−0.070	0.012	−0.295	<0.001	−1.169	0.213	−0.290	<0.001	−0.861	0.208	−0.221	<0.001
Adjusted R-squared	0.170				0.127				0.107			

B: partial regression coefficients, SE: standard error, beta: standardized partial regression coefficients. ^a^ The log-transformed values were used in the analysis.

## Data Availability

The data that support the findings of this study cannot be publicly disclosed for ethical reasons. If you are a researcher who is interested in using this data, please request access to confidential data from the Fukushima Medical University Ethics Committee (rs@fmu.ac.jp).
